# Comparison of the Fungal Community, Chemical Composition, Antioxidant Activity, and Taste Characteristics of Fu Brick Tea in Different Regions of China

**DOI:** 10.3389/fnut.2022.900138

**Published:** 2022-05-17

**Authors:** Yulian Chen, Jiaxu Chen, Ruyang Chen, Leike Xiao, Xing Wu, Lin Hu, Zongjun Li, Yuanliang Wang, Mingzhi Zhu, Zhonghua Liu, Yu Xiao

**Affiliations:** ^1^College of Food Science and Technology, Hunan Agricultural University, Changsha, China; ^2^College of Animal Science and Technology, Hunan Agricultural University, Changsha, China; ^3^Longping Branch Graduate School, Hunan University, Changsha, China; ^4^Key Laboratory of Ministry of Education for Tea Science, College of Horticulture, Hunan Agricultural University, Changsha, China; ^5^National Research Center of Engineering Technology for Utilization of Botanical Functional Ingredients, Hunan Agricultural University, Changsha, China

**Keywords:** Fu brick tea, fungal community, chemical compositions, antioxidant activity, taste characteristics

## Abstract

In this study, the fungal community structure, metabolites, antioxidant ability, and taste characteristics of five Fu brick tea (FBT) from different regions of China were determined and compared. A total of 69 operational taxonomic units (OTUs) were identified and assigned into 5 phyla and 27 genera, with *Eurotium* as the predominant genus in all samples. Hunan (HN) sample had the strongest fungal diversity and richness, followed by Guangxi (GX) sample, and Zhejiang (ZJ) sample had the lowest. GX sample had higher amounts of gallic acid (GA), total catechins, gallocatechin (GC), and epicatechin gallate (ECG) as well as antioxidant activity than the other samples. The levels of total phenolics, total flavonoids, epigallocatechin (EGC), catechin, epicatechin (EC), thearubigins (TRs), and theaflavins (TFs) were the highest in the ZJ sample. Guizhou (GZ) and Shaanxi (SX) samples contained the highest contents of epigallocatechin gallate (EGCG) and gallocatechin gallate (GCG), respectively. Total phenolics, GA, EC, CG, and TFs were positively associated with most of fungal genera. Total phenolic content (TPC), total flavonoid content (TFC), and most of catechins contributed to the antioxidant activities of FBT. HN sample had the strongest sourness and sweetness, ZJ sample had the strongest saltiness, SX sample had the strongest umami, and GZ sample had the strongest astringency, which was ascribed to the varied metabolites. This work reveals that FBT in different regions vary greatly in fungal community, metabolites, antioxidant activity, and taste characteristics, and provides new insight into the quality characteristics formation of FBT in different regions.

## Introduction

**FIGURE 1:**
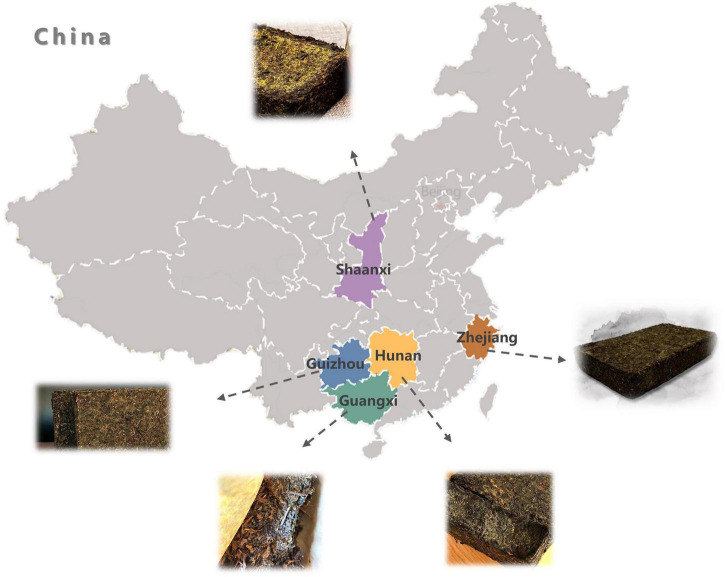
The five Fu brick teas were collected from different provinces of China.

Conventional tea can be traditionally categorized into six types according to oxidation and fermentation degree: non-oxidized green tea, slightly oxidized white tea, lightly oxidized yellow tea, partially oxidized oolong tea, fully oxidized black tea, and post-fermented dark tea. As one of the important commercial dark tea varieties, Fu brick tea (FBT) is a special post-fermented dark tea manufactured from raw matured leaves of *Camellia sinensis* var. *sinensis* through the following steps: steaming, piling, tea brick pressing, fungal fermentation, and drying (1), in which fungal fermentation played a critical role in the formation of its quality characteristics. FBT has attracted people’s interest throughout China, Korea, and Japan due to its unique sensory attributes and health-promoting effects, including anti-obesity, anti-hyperlipidemic, anti-proliferative, and antidysenteric effects (2–4). The main production regions of FBT are Shaanxi (SX) province, Hunan (HN) province, Guizhou (GZ) province, Zhejiang (ZJ) province, and Guangxi Zhuang Autonomous Region (GX) in China (Figure 1), especially in Hunan and Shaanxi provinces.

The special taste and health benefits of FBT are closely associated with the formation of metabolites during fungal fermentation. Fungal community and its relationship to metabolites formation have been previously investigated (5–8). In the past few years, the biotransformation of chemical constituents related to FBT quality during microbial fermentation has been identified; these constituents include free amino acids, polyphenolic compounds (phenolic acids, catechins and their derivatives, flavonoids, and tea pigments, etc.), purine alkaloids, and terpenoids (9). They are formed due to a sequence of biochemical reactions including oxidation, structural modification, methylation, degradation, condensation, and glycosylation catalyzed by extracellular enzymes from functional core microorganisms (10). Previous studies have revealed that *Eurotium*, *Aspergillus*, and *Penicillium* are the dominant genera during FBT manufacturing (11). The raw materials, processing and cultivation conditions of diverse FBT during manufacturing are different, which might greatly influence their microbial composition and caused the different quality characteristics. It was previously reported that the fungal community composition of various FBT vary greatly (12), which might lead to the great differences of chemical components in FBT. Different filamentous have significant effects on transformation ability of phenolic acid, catechines, flavonoids, and anthocyanins. For instance, both *A. niger* and *E. cristatum* reduces galloyl catechins and increase theabrownins (TBs), while *E. cristatum* contribute to the accumulation of B-ring fission derivatives of degalloyl catechins (13) and *A. niger* contribute to the accumulation of A-ring fission derivatives of degalloyl catechins in the fermentation (9). Therefore, it is of significant importance for analyzing the fungal community to enhance our understanding of the mechanisms on quality characteristics formation of FBT in different regions. High-throughput sequencing is a rapid, sensitive, and comprehensive technique that provides thousands of reads in a run and accurately describes microbial diversity in food matrices, which received great attention in the last years and become the main method to analysis of microbial composition. However, systematic study on the fungal community by using this approach, and analyze its influence on the metabolites formation of FBT in different regions of China is still lacking.

Numerous literatures have concentrated on the *in vitro* antioxidant activities of FBT and its association with chemical metabolites (14–16). It is suggested that the antioxidant capacity is recently attributed to flavonols and flavones, tea pigments, caffeine, alkaloids, terpenoids, polysaccharides as well as catechins and their derivatives (3, 14–16). For instance, tea catechins, especially epigallocatechin gallate (EGCG), have been extensively investigated for their excellent antioxidant activity. Likewise, both kaempferol-3-O-glucoside in flavonoids and TBs in tea pigments have significant impact on antioxidant capacity (14). Moreover, it is of great importance to mention that the antioxidant activity in FBT has been reported to be superior compared with other teas (16). FBT in different regions might have diverse antioxidant capacity due to their different metabolites, which have not yet been clearly studied. In addition, previous studies reported that FBT shows strong smoothness, sweetness, and mellowness attributes but with low levels of bitter and astringent. The distinctive taste phenotype of FBT is caused by differences in the contents of various metabolites (5, 8, 12). For instance, catechins, flavonols, and flavones and caffeine are key contributors to the bitterness and astringency of FBT infusion, which greatly influences tea quality (8, 12). The taste profile was mainly detected by an E-tongue sensor system, which simulates human tongue and exhibits good reproducibility, high sensitivity, and low detection limits of tastes compared with the human sense of taste (17). As an emerging technique, E-tongue has been increasingly used to describe the taste characteristics of dry-cured pork, fruit juice, and tea infusion (18, 19). To date, detailed and clear data on the diversity of antioxidant activity and taste quality of FBT produced from different regions of China is rare studied, and their association with bioactive metabolites is not available.

Therefore, the present work aims to investigate the structure and diversity of fungal communities by using Illumina Miseq sequencing of ITS2 region and illustrate the function of microorganisms in the conversion of chemical constituents. Network correlation analysis was used to evaluate the effect of metabolites on the antioxidant activity *in vitro*. The E-tongue system was also employed to detect the taste attributes of five FBT and clarify the key chemical substances contributing to variations in the taste profile. This study is of significant importance for providing valuable information to enhance our understanding of the mechanisms on quality characteristics formation of FBT in different regions of China.

## Materials and Methods

### Materials and Chemical Reagents

Catechin, epicatechin gallate (ECG), EC epicatechin (EC), epigallocatechin (EGC), gallocatechin (GC), catechin gallate (CG), EGCG, and gallocatechin gallate (GCG) were acquired from Sigma-Aldrich Co. (St. Louis, MO, United States). High-Performance Liquid Chromatography (HPLC)-grade methanol was obtained from Merck (Darmstadt, Germany). All other chemicals and reagents were of analytical grade. FBT is mainly consumed in Shaanxi province, Hunan province, Guizhou province, Zhejiang province, and Guangxi Zhuang Autonomous Region in China. From these main producing areas, five FBT samples were collected from large factories and named as SX (Shaanxi Xianxi Lamu Tea Co., Ltd.), HN (Anhua Yuntiange Tea Industry Co., Ltd.), GZ (Guizhou Fanjin Tea Industry Co., Ltd.), ZJ (Zhejiang Wuyi Luotuo Jiulong Brick Tea Co., Ltd.), and GX (Guangxi Jinhua Tea Co., Ltd.). All tea samples used in this study were produced in 2018.

### DNA Extraction and ITS2 Sequencing

Microbial genomic DNA was extracted from FBT samples by using E.Z.N.A. Stool DNA Kit (D4015, Omega, Inc., United States) based on the manufacturer’s instructions. Nuclear-free water was used for blank and elution buffer (50 μL) was used to elute the total DNA. The ITS2 gene was amplified by PCR with forward primers fITS7 (5′-GTGARTCATCGAATCTTTG-3′) and ITS4 (5′-TCCTCCGCTTATTGATATGC-3′). The amplification process was conducted in a reaction mixture (25 μL) with 2.5 μL of each primer, 12.5 μL PCR Premix, 25 ng of template DNA, and PCR-grade water to adjust the volume. The PCR conditions were as follows: initial denaturation for 30 s at 98°C; 35 cycles of denaturation for 10 s at 98°C, annealing for 30 s at 54°C/52°C, and an extension for 45 s at 72°C; and final extension for 10 min at 72°C. The PCR products were extracted from 2% agarose gel, purified by AMPure XT beads (Beckman Coulter Genomics, Danvers, MA, United States), and quantified by Qubit (Invitrogen, United States). After sequencing with the amplicon pools, the size and quantity of the amplicon library were estimated on Agilent 2100 Bioanalyzer (Agilent, United States) and with the Library Quantification Kit for Illumina (Kapa Biosciences, Woburn, MA, United States), respectively. Combined PhiX Control library (v3) (Illumina) and amplicon library (expected at 30%) together. The libraries were sequenced in 250PE MiSeq runs, and one library was sequenced using standard Illumina sequencing primers by both protocols without the need for a third (or fourth) index read.

### Determination of Total Phenolic Content, Total Flavonoid Content, Theaflavins, Thearubigins, and Theabrownins

Total phenolic content in tea samples was determined using Folin-Ciocalteu method in accordance with literature (20). The content was calculated based on standard curve with gallic acid (GA) and expressed as milligram of GA equivalent per gram of tea sample (mg GAE/g d.w.). Total flavonoid content was evaluated using aluminum nitrate colorimetry in accordance with the method of Xiao et al. (21). The result was calculated based on standard curve with rutin and expressed as grams of rutin equivalent per gram of dry tea sample (g RE/g d.w.). The contents of TFs, TRs, and TBs in the tea samples were systematically measured using the method in literature (22).

### Determination of Gallic Acid and Catechins by High-Performance Liquid Chromatography

FBT samples were freeze-dried and crushed using an electric grinder (FW 100, Beijing Yongguangming Medical Instrument Co., Ltd., Beijing, China). About 1 g of tea sample was placed in an extraction bottle and added with deionized water [1:40 (w/v)]. The sample was subjected to thermal extraction at 95°C for 30 min, with shaking every 5 min. The blend was centrifuged for 15 min at 10,000 × g after cooling, and the supernatant was collected. After the volume was adjusted to 50 mL, the sample was stored in darkness for future use.

The contents of catechin, GA, GCG, EGC, GC, EC, EGCG, ECG, and CG in the tea samples were analyzed using HPLC (23). A 0.45 μm PVDF membrane was used to filter the sample extracts. The filtrate (10 μL) was added into the Agilent 1260 Infinity II LC system (Agilent, Santa Clara, United States) for analysis of GA and catechins. An Agilent 5 TC-C18 (2) reverse-phase column (Eclipse Plus, Agilent Technologies, United States) (5 μm particle size; 4.6 mm × 250 mm) was also used. Water containing 0.1% (v/v) formic acid (mobile phase A) and acetonitrile (mobile phase B) were used for chromatographic elution as follows: 0–40 min, 10–35% B; 40–42 min, 35–10% B. The flow rate was kept at 0.8 mL/min for a total running time of 42 min at 25°C. The sample was monitored at 210 nm and subjected to a photodiode array detector (Agilent G7114A variable wavelength detector; Agilent, Santa Clara, United States). The identification and quantification of catechin, GA, GCG, EGC, GC, EC, EGCG, ECG, and CG were performed by comparing their UV spectra and retention times with their authentic standards. The contents were calculated as mg/100 g of dry weight tea samples. Total catechins were the summation of GCG, catechin, EGC, GC, EC, EGCG, ECG, and CG contents.

### Evaluation of Antioxidant Activities

Several assays with different mechanisms were conducted to assess the antioxidant activities of the FBT samples. ABTS radical cation scavenging activity (ABTS), DPPH radical scavenging activity (DPPH), reducing power (RP), and hydroxyl radical scavenging ability (HAS) were performed according to our previous study (24). An array of vitamin C concentration were carried out to plot the calibration curve, and the results were expressed as milligram of vitamin C per gram dry weight FBT flour (mg VCE/g d.w.). Ferric reducing antioxidant power (FRAP) was conducted using the method reported by Xiao et al. (20). Various concentrations of FeSO_4_ solution were used to plot the calibration curve. The FRAP values were presented as millimole Fe (II) equivalents per gram dry weight sample.

### Electronic Tongue Analysis

The electronic tongue analysis of FBT samples from different regions in China was performed using TS-sa402b (INSENT Inc., Japan), which has imitated lipid membrane sensors with a wide area of selection. Every sample was infused with 200 mL of distilled boiled water (40°C) to homogenize (10,000 r/min) for 1 min. The tea infusion was then centrifuged at 3,000 rpm for 10 min at 4°C, and the supernatant was filtered through three layers of gauze. The determination process contained three steps: 30 s of sample detection, 30 s of aftertaste detection and 120 s of cleaning. Each FBT infusion was detected four times, and the average value of the last three cycles was determined, which were analyzed using TS-sa402b Library search software (INSENT, Japan) to characterize the taste quality of FBT samples.

### Statistical Analysis

All measurements were conducted in triplicate, and data were presented as mean value ± standard deviation (*SD*) and analyzed by ANOVA. The significance of differences (*p* < 0.05) between the average values was determined by Duncan’s multiple range test using SPSS version 16.0 (SPSS Inc., Chicago, United States). Data graphs were plotted by OriginPro 8.1 statistical software (OriginLab Co., United States). With regard to the ITS2 sequencing data, mixed sample amplicons were paired-end sequenced on an Illumina MiSeq platform according to the manufacturer’s protocol from LC-Bio. Raw tags were quality-controlled filtered under specific conditions using fqtrim (V 0.94). Chimeric sequences were filtered using Vsearch software (v2.3.4). The representative sequences of operational taxonomic units (OTUs) were obtained by clustering ≥97% similarity using Vsearch (v2.3.4). Alpha diversity was determined by Chao1, Observed species, Goods coverage, Shannon, and Simpson indices using QIIME (Version 1.8.0). Beta diversity was estimated using (PCoA) and clustered using QIIME software (Version 1.8.0).

## Results and Discussion

### Comparison of Fungal Communities of Fu Brick Tea Produced in Different Regions

#### Diversity of Fungal Communities

**FIGURE 2:**
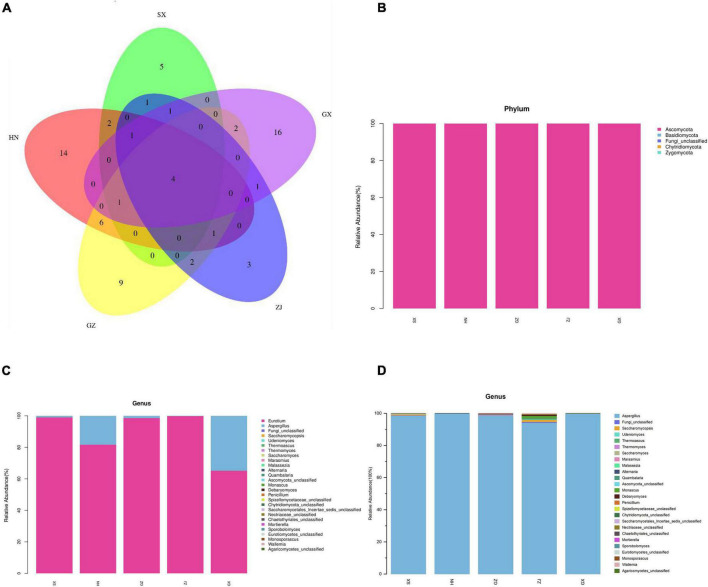
The Venn diagram analysis of fungal community compositions of five Fu brick teas in different regions **(A)**. The average relative abundance of fungal communities **(B)** at phylum level; **(C)** at genus level; **(D)** genus level with the highest abundance group removed] of FBT samples.

A total of 1,667,155 effective reads were generated from the five FBT samples in quadruplicate analysis after filtering quality and removing chimera, with a mean sequence number of 83,358 for each sample (20 samples; 79,869–86,727 reads per sample). On the basis of the 97% similarity threshold, these high-quality sequences were clustered into 69 OTUs for all samples, from the range of 4–19 OTUs per sample. The rarefaction curves for Shannon diversity reached saturation (Supplementary Figure 1), suggesting that the sequencing dataset was adequate to distinguish the species richness in the five regional FBT samples. The diversity and richness of the fungal communities was measured by alpha diversity analysis using ACE, Chao1 (community richness), goods coverage (sequencing depth), Shannon (evenness), and Simpson (richness) indices (Supplementary Table 1). The goods coverage of all the samples was >99.99%, confirming that the sequencing depth was sufficient to saturate the fungal diversity and most fungal microbes had already been captured, consistent with the rarefaction curves. As shown in Supplementary Table 1, no significant differences were observed in the Shannon and Simpson indices (the diversity of detected fungal community) among SX, GZ, and ZJ samples and between HN and GX samples (*p* > 0.05). The fungal diversity of HN and GX samples was higher than that of SX, GZ, and ZJ samples. In addition, the ACE and Chao1 indexes of the HN sample was higher than those of the other samples, consistent with the differences in the observed OTUs and indicating HN sample shows the highest fungal richness. The results implied that the fungal diversity and richness of the HN sample was the best, followed by GX, and the ZJ sample was the lowest. Rank-abundance curves revealed that a large proportion of the sequences were classified as rare microorganisms represented by only a few sequences, especially for ZJ sample (Supplementary Figure 2). The beta diversity results of NMDS analysis were analyzed based on unweighted uniFrac to estimate the distribution of fungal communities in the five FBT samples. As shown in Supplementary Figure 3, the fungal community structure of the GX sample had the highest level of similarity, while the GZ sample had the lowest. Venn diagram analysis was used to present the number of features that are common and unique to each sample/group of different FBT samples. Only 4 of the identified OTUs were shared in all groups. Eight of 36 OTUs, 6 of 49 OTUs, 6 of 37 OTUs, and 12 of 42 OTUs were shared between the HN sample and the SX, GX, ZJ, and GZ samples, respectively. Six of 34 OTUs, 7 of 22 OTUs, and 5 of 35 OTUs were shared between the SX sample and GX, ZJ, and GZ samples, respectively. Seven of 33 OTUs and 7 of 44 OTUs were shared between the GX sample and ZJ and GZ samples, respectively. Seven of 32 OTUs were shared between ZJ and GZ samples (Figure 2A). These results clearly demonstrated that fungal communities of FBT produced in different regions are greatly different.

#### Composition of Fungal Communities in Fu Brick Tea

**FIGURE 3:**
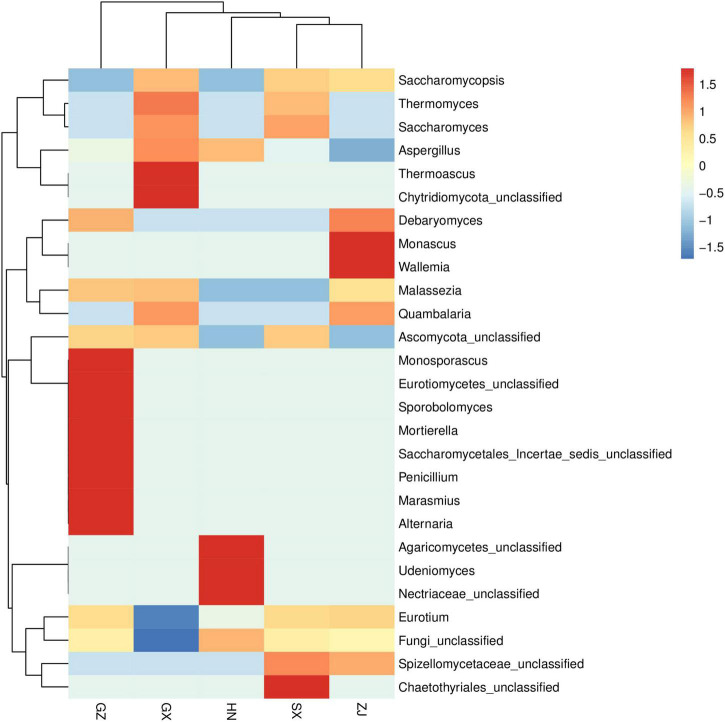
Cluster analysis on fungal genera in five Fu brick teas in different regions.

**FIGURE 4:**
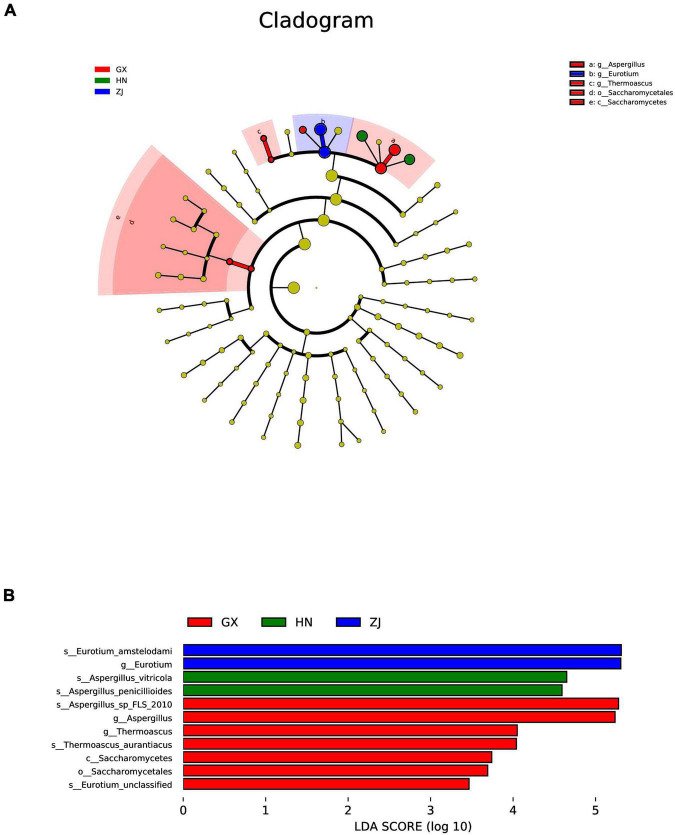
Cladogram **(A)** and linear discriminant analysis (LDA) score **(B)** showed the significant abundance differences of fungal taxa by LEfSe analysis. The node size represents the difference in relative abundance.

Ribosomal Database Project (RDP) and unite databases were used for species classification and subsequent analysis to ensure the complete and accurate annotation results at the 70% threshold to investigate the taxonomic composition of microbial community and diversity among the five FBT samples. The detected fungal communities were subdivided into 5 phyla, 15 classes, 20 orders, 23 families, 27 genus, and 33 species. As the taxonomical granularity increased, the percentages of unclassified fungal ITS rRNA gene sequences increased to 1.86 at the species level. As shown in Figure 2B, Ascomycota was the only predominant phylum in all sequences, making up 99.97–100% of the total relative abundance. Basidiomycota, Chytridiomycota, and Zygomycota, even if they were also detected, accounted for a low percentage of the total effective sequences. At the genus level (Figure 2C), *Eurotium* and *Aspergillus* had more than 99.97% of all valid reads. *Eurotium* was predominant in all samples and represented the largest fraction, with average relative abundance of up to 88.84%, consistent with previous reports (24, 25). *Eurotium* is widely regarded as the main fungus in the fermentation of FBT (26), which has been reported as the key contributor in the formation of the special chemical components of FBT (5). *E. cristatum* could secrete many extracellular enzymes that have the great potential to transform chemical compounds in tea leaves into other components. For instance, previous studies reported that *E. cristatum* played an important role in the significant increase of TBs, soluble carbohydrates and three purine alkaloids (27). In addition, *E. cristatum* transformed phenolic compounds that leading into stronger red and yellow color of tea infusion (28). When the fungal composition was analyzed at the genus level with the dominant group removed (Figure 2D), *Aspergillus* was overwhelmingly dominant in all samples (SX 98.55%, HN 99.81%, GZ 98.59%, ZJ 94.17%, and GX 99.91%, respectively). *Aspergillus* is a genus of filamentous and saprophytic fungi that are widely distributed in nature and contains more than 250 species (29). Numerous previous studies have indicated that the quality formation of FBT was also closely related to the metabolism of *Aspergillus* genus. For instance, Zhao et al. reported that *Aspergillus* produced a variety of enzymes (pectinesterase, endo-1,4-β-xylanase, catalases, catalase-peroxidases, alkaline protease, and peroxiredoxins) that contributed to the oxidation of tea polyphenols and degradation of plant cell wall (30). Furthermore, the genus *Aspergillus* make great contribution to the enhancement of taste by secreting enzymes like proteases, α-amylases glucoamylases, xylanases, and other hemicellulose to decompose complex carbohydrates, proteins and lipid into small molecules in FBT and produce unique flavor (31, 32). Besides, it was found that *Saccharomycopsis* and *Debaryomyces* were more abundant in the ZJ sample than that in other regions, and *Monascus* and *Wallemia* were the two genera that only existed in the ZJ sample. Among them, the genus *Debaryomyces* has been considered to be an essential candidate microorganism in a fermentation starter for artificial fermentation of FBT, as it has a distinguished influence on the metabolites biotransformation during the manufacturing process. Their enzyme activities enhance the quality of FBT by producing the sweet substance xylitol, vitamins, and other organic acids, contributing to richer taste and flavor (33). Some species of *Monascus* genus have been widely used in tea fermentation owing to their abilities to decrease hydrogen peroxide and to bio-convert tea polyphenols into TFs and TRs during the fermentation process, exhibiting a strong antioxidant activity by preventing oxidative stress-induced diseases (34). The genus *Wallemia* was also identified as the core functional microorganisms that associated with the metabolic variations of volatile components during the pile-fermentation of FBT, and may play an important role in contributing the stale and betelnut aroma in dark tea (35). To better understand differences and similarities among different samples, we carried out Z-value transformation on the basis of the heat map and did cluster analysis on fungal genera (Figure 3). Among the 27 detected genera, eight were found in the GZ sample but not in the other samples. These unique genera were *Marasmius*, *Alternaria*, *Penicillium*, *Saccharomycetales Incertae sedis* unclassified, *Mortierella*, *Sporobolomyces*, *Eurotiomycetes* unclassified, and *Monosporascus*. Only one unique genus was found in the SX sample, namely, *Chaetothyriales* unclassified, and it also had the least number of genus-level community composition. LEfSe analysis was carried out to determine the specific fungal taxa that can distinguish FBT samples from different regions. LDA emphasized that about 11 fungal clades, whose abundance in a given regional variety differed from those in the other samples, exhibited statistically significant differences (Figure 4A). LEfSe was used to illustrate differentiating taxa on the phylogenetic tree of the fungal community in the FBT samples from the domain to the species level (Figure 4B). No distinguishable fungal taxa were detected in GZ and SX samples. However, three residual samples appeared as differentially abundant fungi belong to one class (Saccharomycetes), one order (Saccharomycetales), 3 genera (*Eurotium*, *Aspergillus*, *Thermoascus*), and 6 species (*Eurotium amstelodami*, *Aspergillus vitricola*, *A. penicillioides*, *A.* sp. FLS 2010, *Thermoascus aurantiacus*, and *Eurotium* unclassified). The GX sample had the largest number of discriminate taxa, involving class Saccharomycetes, order Saccharomycetales, and genera *Aspergillus*, *Eurotium*, and *Thermoascus*, which were all statistically different and distinctive. *Eurotium* and *Aspergillus* were significantly enriched in HN and ZJ samples, respectively.

### Main Metabolites of Fu Brick Tea From Different Regions

**FIGURE 5:**
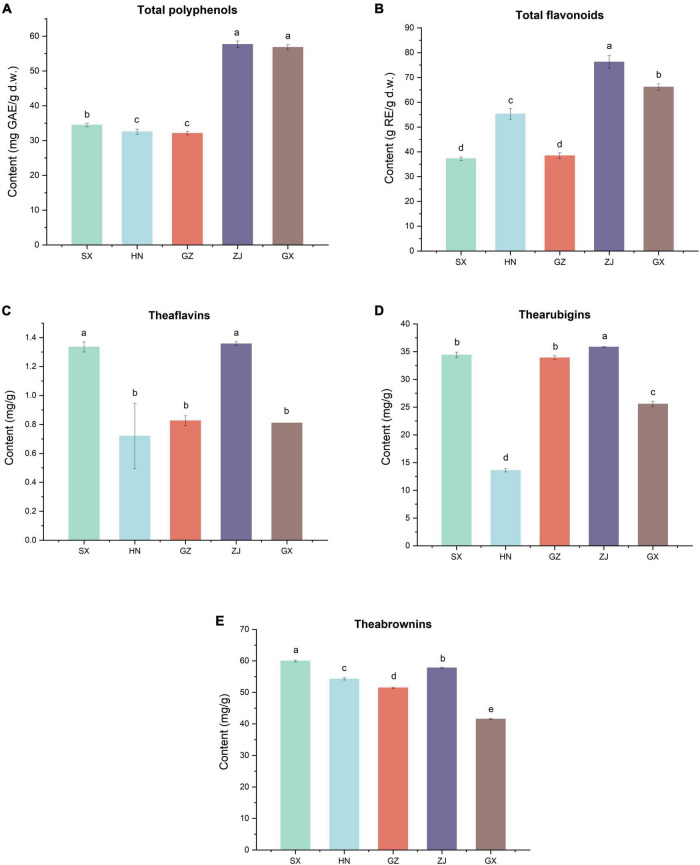
Contents of **(A)** total polyphenols, **(B)** total flavonoids, **(C)** theaflavins, **(D)** thearubigins and **(E)** theabrownins of five Fu brick teas in different regions.

**FIGURE 6:**
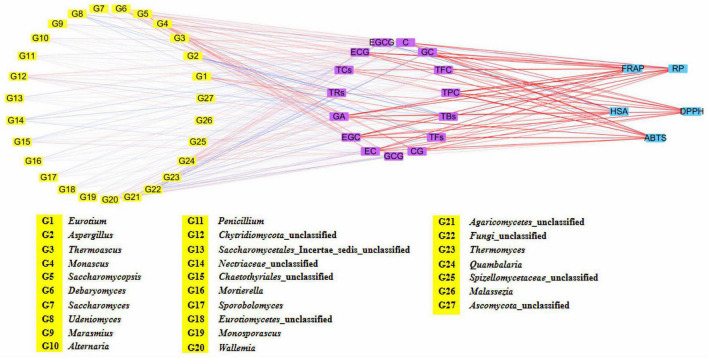
Network visualized the relationship between main metabolites and fungal community, as well as antioxidant activity and main metabolites of five Fu brick teas in different regions.

The TPC and TFC of the five FBT samples from different regions were evaluated (Figures 5A,B). The TPC and TFC in the ZJ sample were the highest (57.64 mg GAE/g d.w. and 76.24 g RE/g d.w., respectively). The GZ sample had the lowest TPC and TFC, which were 1.80 and 1.99 times lower than those in the ZJ sample. The HN sample contained higher TFC (55.28 g RE/g d.w.) and lower TPC (32.54 mg GAE/g d.w.) than the SX sample. The different results of FBT samples were partly due to their diverse fungal community composition. In this study, network correlation analysis revealed that TPC and TFC were significantly positively associated with *Monascus*, *Debaryomyces*, *Wallemia*, and *Quambalaria.* TFC was also found to be strongly correlated with *Monascus*, *Wallemia*, *Thermomyces*, and *Quambalaria* (Figure 6). Previous studies have also reported that the TPC was highly positively related to *Cyberlindnera*, *Wallemia*, *Penicillium*, *Ascomycota* unclassified, and *Eucasphaeria*, while negatively correlated with *Aspergillus* during manufacturing process of FBT (36). The TFC were significantly positively (*p* < 0.05) associated with *Candida*, *Cyberlindnera*, *Debaryomyces*, and *Penicillium*, while were negatively correlated with *Chlamydomonas* during the pile-fermentation period (37). Changes of phenolics and flavonoids are connected with the action of extracellular microbial enzymes, such as glucansucrase and cellulase, secreted by microorganisms including *Chlamydomonas*, *Penicillium*, and *Aspergillus* (9). Moreover, *A. pallidofulvus* and *A. sesamicola* make contribution to the accumulation of several flavonoids during fermentation (38, 39). It has been illustrated that the genus *Debaryomyces* increase both TPC and TFC during pile-fermentation probably attributed to the tannase secreted by the microbe, which could hydrolyze the ester and phenol condensation bonds in gallic tannin to produce simple catechins and GA, and also virtually hydrolyze the acetal bonds of EGCG to produce GA and EGC (40). The *Thermomyces* genus was reported to be one of the high-temperature resistant genera and occur when the humidity and temperature sharply increased during the early and mid-periods of pile-fermentation. Three non-volatile compounds (L-Tyrosine, L-Phenylalanine and GA) and six volatile compounds (2-ethylhexanol, rose oxide, safranal, α-ionone, β-Ionone, and ionone) were found to be significantly or highly significantly positively correlated with *Thermomyces*, contributing to the unique taste and flavor of dark tea (37, 41). However, the TFC in FBT declined significantly during manufacturing (36), which could be caused by moisture and heat reaction apart from the influence of microorganisms. The quality of FBT was mainly affected by raw materials and processing technology. Nevertheless, the degradation of TPC, TFC, and flavonoid glycosides during microbial fermentation might contribute to the taste characteristics and special aroma of dark tea (42).

As the main component of tea polyphenols (accounting for approximately 60–80%), catechins are divided into ester and non-ester catechins, and the molecular structure of the former has 1–2 more galloyl groups than the latter, and their content has a certain influence on the characteristic flavor and bioactivities of teas (9). The galloyl moiety of catechins is an essential donor to strong bitterness and astringency, while non-gallated catechins are not astringent (43). Therefore, GA, EGC, GC, EC, catechin, GCG, EGCG, CG, and ECG were identified and quantified by HPLC analysis to better understand the difference in the GA and catechins content of FBT produced from different regions (Table 1). The level of GA in the GX sample was significantly higher than that in the other samples (*p* < 0.05). GA and its derivatives are abundant in dark tea and exhibit anti-inflammatory, anti-mutagenic, and antioxidant activities as well as cardiovascular protective effects (44). In the present research, GA was positively associated with *Monascus*, *Debaryomyces*, *Wallemia*, and *Quambalaria* (Figure 6). Previous studies reported that the formation of GA probably resulted from moisture and heat reaction in the early stage and microbial metabolism is the main contributor at the later stage (33) during FBT manufacturing. In the early stage, the galloylated catechins underwent a gradual decomposition through hydrolysis by the effect of moisture and heat treatment, then corresponding degalloyl catechins along with GA were produced (28). Among them, EGCG is one of the most abundant and active galloylated catechins and was reported to be the main pathway for GA biosynthesis (45). In addition, the microbial tannase contributed to the formation of GA through the degradation of gallotannins like 3-O-galloylquinic acid or 1-galloylglucose and 1,2-digalloylglucose (6). Zhao et al. found that the content of EGCG and ECG decreased from 47.3 ± 2.42 and 39.94 ± 2.59 mg/g to 0.08 ± 0.15 and 0.48 ± 0.3 2 mg/g, respectively, after fermentation (30). By contrast, they found that the level of GA increased more than 11 times. Except for GA, the contents of total catechins, GC, and ECG were the highest in the GX sample. Tea samples produced from GZ and SX had the highest contents of EGCG (690.3 ± 0.89 mg/100 g) and GCG (82.48 ± 2.68 mg/100 g), respectively. The ZJ sample had higher amounts of non-galloylated catechins, including EGC, catechin, and EC, which could be attributed to the hydrolysis of galloylated EGCG, CG, and ECG; the lower bitterness and astringency in the ZJ sample could be explained at the same time (46). Interestingly, our results showed that GA, EGC, GC, EC, catechin, CG ECG, and total catechins were closely correlated with *Quambalaria* (Figure 6). As shown in Figure 6, catechin was positively related to the genus *Debaryomyces*, *Monascus*, *Wallemia* and *Quambalaria*. EGCG was significantly positively correlated with *Alternaria*, *Penicillium*, *Marasmius*, *Udeniomyces*, etc. ECG was positively associated with *Thermoascus*, *Saccharomycopsis*, *Saccharomyces*, *Thermomyces*, *Quambalaria*, etc. This result was comparable to the results from previous studies. For instance, it has been reported that the catechin was positively associated with *Candida*, *Cyberlindnera*, *Debaryomyces*, and *Penicillium* (37). ECG was significantly positively correlated with *Candida*, *Wallemia*, *Penicillium*, and *Psychrobacter*, while negatively correlated with *Carnobacterium* and *Aspergillus* (36). EGCG was significantly associated with *Penicillium* and *Alternaria*, while negatively associated with *Burkholderia-Caballeronia-Paraburkholderia* and *Aspergillus* (12, 36, 47). GCG was significantly correlated with *Wallemia*, *Penicillium*, *Cyberlindnera*, and *Candida* (36). During microbial fermentation process, the formation of GA and non-gallyolated catechins are resulted from the gradual hydrolysis of gallyolated catechins and polymeric catechins in FBT. At the same time, non-galloylated catechins are further degraded into phenolic acids or converted into complex phenolic tea pigments under the chemical reactions, such as oxidation and polymerization, leading to their decrease (48). For this reason, several catechin derivatives were newly formed in FBT, involving 2S, 3R-6-methoxycar-bonylgallocatechin (MCGE) and 2R, 3R-6-methox-ycarbonylgallocatechin-3-O-gallate (EGCGD) (4). Besides, due to the strong microbial action and hydrolysis during the fermentation of FBT, chemical reactions, such as oxidation and isomerization of catechins, occur, and a large amount of ester catechins are degraded into simple catechins. Larger amount of non-ester catechins to ester catechins leads to less astringent and more mellow taste (49).

**TABLE 1:** Contents of gallic acid and catechins of five Fu brick teas in different regions.

Compounds	Contents (mg/100 g)				
	SX	HN	GZ	ZJ	GX
GA	181.5 ± 1.37^c^	123.0 ± 2.10^d^	117.3 ± 2.75^d^	310.2 ± 4.47^b^	416.4 ± 4.47^a^
GC	113.0 ± 2.06^c^	19.61 ± 1.28^d^	114.7 ± 0.97^c^	171.4 ± 2.95^b^	190.0 ± 2.19^a^
EGC	60.67 ± 0.63^c^	7.03 ± 2.21^d^	179.9 ± 0.46^b^	574.6 ± 4.93^a^	575.3 ± 6.18^a^
Catechin	17.01 ± 0.22^c^	16.49 ± 0.55^c^	10.68 ± 2.15^d^	40.63 ± 0.03^a^	28.86 ± 1.40^b^
EC	25.48 ± 0.26^e^	29.19 ± 0.42^d^	53.31 ± 0.77^c^	246.7 ± 0.66^a^	163.4 ± 0.56^b^
EGCG	58.63 ± 1.13^e^	664.9 ± 1.91^b^	690.3 ± 0.89^a^	134.7 ± 1.58^d^	403.5 ± 5.61^c^
GCG	82.48 ± 2.68^a^	9.41 ± 1.82^d^	35.63 ± 0.81^c^	38.20 ± 0.44^c^	71.78 ± 3.52^b^
ECG	12.65 ± 0.35^d^	5.88 ± 0.09^e^	24.64 ± 0.44^c^	74.60 ± 2.33^b^	115.0 ± 2.79^a^
CG	9.31 ± 0.25^c^	10.03 ± 0.44^c^	6.38 ± 0.18^d^	67.90 ± 0.62^a^	18.92 ± 0.39^b^
Total catechins	379.2 ± 2.63^e^	762.5 ± 4.90^d^	1115.5 ± 3.21^c^	1348.7 ± 4.30^b^	1566.8 ± 11.47^a^

*GA, gallic acid; GC, gallocatechin; EGC, epigallocatechin; EC, epicatechin; EGCG, epigallocatechin gallate; GCG, gallocatechin gallate; ECG, epicatechin gallate; CG, catechin gallate. Each tea sample was determined with three replications. Letters indicate Duncan’s pairwise differences among different samples (p < 0.05).*

Tea pigments such as TBs, TFs, and TRs are the water-soluble oxidation products of polyphenols in tea infusion and significantly contribute to the characteristic taste and color of FBT (46). TFs are primary transitional metabolites, which have been presumed to be produced initially by the oxidative polymerization of tea polyphenols, especially catechins, and were further polymerized into TBs with other compounds (such as polysaccharides, proteins, and caffeine) during pile fermentation (50). The level of TFs was the highest in the ZJ sample (1.36 ± 0.01 mg/g) and was almost equivalent to that in SX sample (1.34 ± 0.03 mg/g). Nevertheless, no statistically significant differences were found in the level of TFs among HN, GZ, and GX samples (Figure 5C). A significant positive correlation (*p* < 0.05) existed between the levels of TFs and the genera of *Eurotium*, *Monascus*, *Debaryomyces*, *Chaetothyriales* unclassified, *Wallemia*, and *Spizellomycetaceae* unclassified (Figure 6). Lee et al. used *Monascus* as the strain to ferment tea for thirty days, the content of TFs in tea increased almost nine times compared with unfermented tea (34). It has also been reported that tea fermentation with inoculated *Debaryomyces hansenii* improved TFs (51). In addition, studies indicated there were other microorganisms including *A. pallidofulvus*, *A. sesamicola*, and *Penicillium manginii* also had significantly (*p* < 0.05) positive influence on TFs (10). Although the content of TFs in tea leaves is lower than that of TRs and TBs, it is an important factor that affects the brightness, taste intensity, and freshness of tea infusion. The ZJ sample also contained the highest level of TRs (35.84 ± 0.11 mg/g), which was 1.44, 1.95, and 10.28 mg/g higher in SX, GZ, and GX samples, respectively. The TRs content (13.60 ± 0.29 mg/g) was the lowest in the HN sample (Figure 5D). The level of TRs was positively associated with *Eurotium* and *Debaryomyces* but negatively correlated with *Aspergillus*, *Udeniomyces*, *Nectriaceae* unclassified, *Agaricomycetes* unclassified, and Fungi unclassified (Figure 6). *A. pallidofulvus* and *A. sesamicola* were also reported to have significant (*p* < 0.05) negative effects on TRs (10). TRs are one of the main substances that contribute to the red color and taste intensity of tea infusion. Importantly, TRs play a role similar to insulin or insulin-like growth factor in human cells and can become a new alternative for the treatment of overweight and obesity; it functions by activating lipolysis via mitochondrial uncoupling capacity and inactivating phosphoenolpyruvate carboxykinase (PEPCK) promoter as the major enzyme of gluconeogenesis through Forkhead box protein O1a (FOXO1a) phosphorylation (52). With the increasing oxidation degree of tea polyphenols, TRs and TFs are further oxidized and polymerized to TBs under the synergistic action of polyphenol oxidase (PPO), peroxidase (POD), and laccase produced by microorganisms, such as the genera *Aspergillus*, *Pseudomonas*, *Eurotium*, *Debaryomyces*, *Lichtheimia*, *Blastobotrys*, *Rosellinia*, *Geotrichum*, *Lichtheimia*, and *Rasamsonia* (9). The content of TBs was the highest in the SX sample (59.96 ± 0.29 mg/g) and was 1.04–1.44 folds higher than that in the four other samples, followed by ZJ sample (Figure 5E). This result was comparable to the results from Wang et al. (53), who determined that the levels of TBs, TFs, and TRs in the fermented tea were 98.67 ± 6.55, 0.98 ± 0.13, and 25.39 ± 10.56, respectively. The TBs content had a strong positive correlation with *Eurotium* but was extremely (*p* < 0.01) negatively correlated with *Thermoascus*, *Chytridiomycota* unclassified, *Thermomyces*, and *Malassezia*. Diverse strains isolated from fermentation of dark tea have been applied for producing TBs involving *E. cristatum, A. niger*, *A. tubingensis*, *A. marvanovae*, *A. fumigatus*, *Rhizomucor pusillus*, and *R. tauricus* (21, 53, 54). *A. sesamicola* was also thought to had highly significant (*p* < 0.01) negative effects on TBs (53). TBs are characteristic constituents in microbial-fermented tea that can regulate fatty acid metabolism and attenuate hypercholesterolemic capacities (55, 56). Previous research confirmed the gradual decrease in tea polyphenols and the accumulation of TBs when catechins were finally converted into TBs during the pile-fermentation process (50). This finding is consistent with the present results that GX sample had the highest TBs content and the lowest total catechin content. In addition, TFs, TRs, and TBs were significantly (*p* < 0.05) negatively associated with *Aspergillus*, with correlation coefficients (*r*) of −0.597, −0.660, and −0.715, respectively, which might be caused by few enzymes discharged from *Aspergillus*; these enzymes include proteases, hemicellulases, α-amylases, and cellulases, which could catalyze tea polyphenols, especially catechins and tea pigments.

### Antioxidant Activities of Fu Brick Tea in Different Regions

**FIGURE 7:**
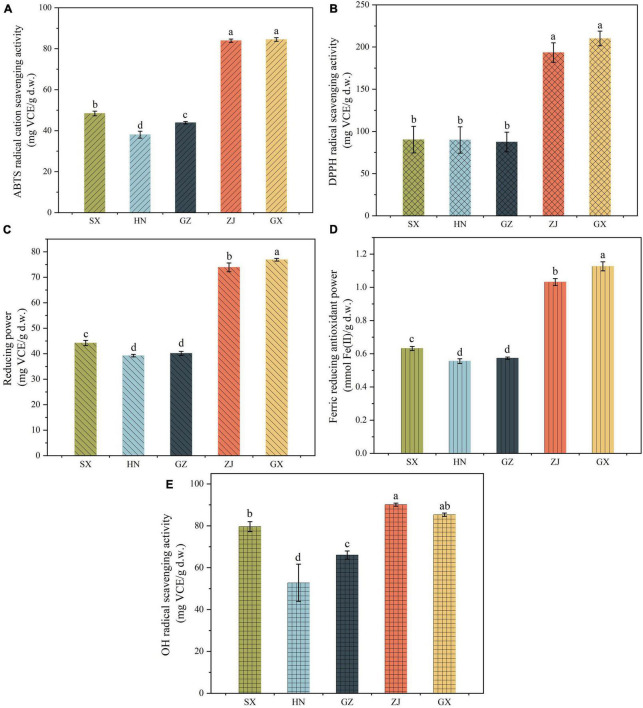
Antioxidant activity of five Fu brick teas in different regions. ABTS radical cation scavenging activity **(A)**, DPPH radical scavenging activity **(B)**, reducing power **(C)**, OH radical scavenging ability **(D)** and ferric reducing antioxidant power **(E)**. Data were recorded as the mean value ± standard deviation of three replicates. Mean values marked by the different letters among the samples denoted significant difference (*p* < 0.05).

**FIGURE 8:**
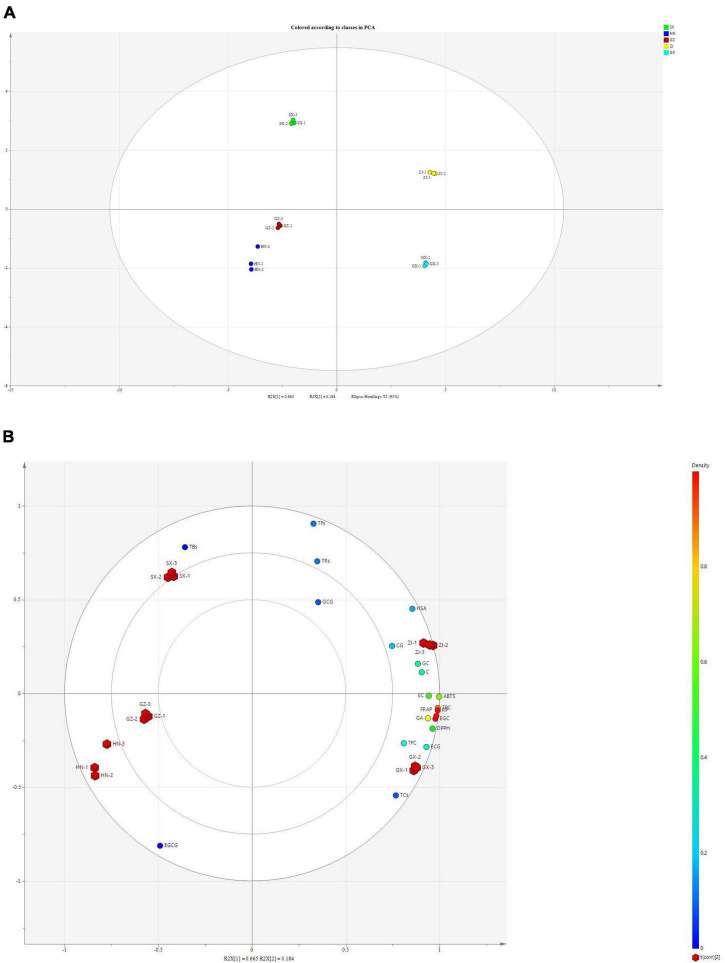
Principal component analysis score **(A)** and loading plot **(B)** assessing the metabolites and antioxidant activity of five Fu brick teas in different regions.

FBT from different regions had diverse *in vitro* antioxidant activities. Five regular antioxidant capacity methods with disparate working mechanisms were used to assess the antioxidant activity of the five FBT samples (Figure 7). The GX sample had relatively stronger antioxidant capacity measured using ABTS (84.50 mg VCE/g d.w.), DPPH (210.14 mg VCE/g d.w.), RP (76.85 mg VCE/g d.w.), and FRAP (1.13 mmol Fe(II)/g d.w.) assays compared with tea samples from the other regions. Despite the lower HSA of GX sample than the ZJ sample, no statistically significant differences were detected among the samples. FBT produced in SX exhibited significant (*p* < 0.05) higher antioxidant activity measured using the five methods than HN and GZ samples. DPPH had no significant (*p* > 0.05) difference between SX and HN samples. The HN sample demonstrated the lowest value in ABTS (38.06 mg VCE/g d.w.), RP (39.24 mg VCE/g d.w.), FRAP (0.56 mmol Fe(II)/g d.w.), and HSA (52.80 mg VCE/g d.w.). Principal component analysis (PCA) was further carried out to detect cluster formation and reveal relationships among tea samples, bioactive composition, and antioxidant properties. As shown in Figure 8A, the PCA score plot indicated that tea samples from different regions were clearly distinguished from one another. The ZJ sample in the upper right part of Figure 8A was positively related to PC1 and PC2. This sample can be considered as having relatively higher antioxidant activity and higher content of polyphenolic metabolites. Loading plots (Figure 8B) were constructed to distinguish the most significant contributors. ABTS, DPPH, RP, and FRAP were found to be in a similar manner loaded on PC1, indicating that the four properties were connected with antioxidant activity. Moreover, most polyphenolic compounds had high loading on PC1 except for EGCG and TBs, which indicated that polyphenols were good antioxidants. According to literature, the reason behind the discrepancy in antioxidant capacity might be based on the presence of polyphenolic constituents, such as tea polyphenols, flavonoids, catechins, TFs, TRs, etc. in different FBT samples (57). Network correlation analysis was conducted to confirm the relationship between the antioxidant activity and polyphenolic components of tea products (Figure 6). The ABTS, DPPH, RP, and FRAP showed highly significant positive correlations with TPC and TFC (| *r*| > 0.805 and *p* < 0.01), while no significant (*p* > 0.05) correlation was found between HSA and TFC. Numerous studies have linked the increase in the antioxidant activity in tea with phenolic compounds (58, 59). The antioxidant activity of phenolic constituents mainly depends on the quantity of OH-groups and their position. The OH-groups on the 3′−, 4′−, and 5′− position of the B-ring of flavonols and flavones enhance the antioxidant capacity of these substances compared with phenolics with one hydroxy group (12). The glycosylation of flavonoids by microbial enzymatic biotransformation during fermentation contributes to the antioxidant activity of FBT. GA and its derivatives have great antioxidant and antimicrobial capacity, which might be due to the methylization and glycosylation of GA by microbial enzymatic activity (60). As predicted, an extremely high correlation was observed between the results obtained of the five antioxidant activity assays involving ABTS, DPPH, RP, FRAP, and HSA and the GA content, with correlation coefficients of *r* = 0.952, *r* = 0.947, *r* = 0.965, *r* = 0.979, *r* = 0.778, respectively (*p* < 0.01). *In vitro* antioxidant activity in microbial enzymatically fermented teas including oolong tea, black tea, dark tea, and white tea was positively related to the high level of catechins (15, 61). All the five antioxidant activity methods exhibited the highly significant (*p* < 0.01) positive correlation with total catechins. Additionally, the antioxidant effects displayed the highest correlation with EGC among all types of catechins (*r* > 0.780). This remarkable antioxidant capacity of EGC can be ascribed to the higher extent of hydroxylation of the B ring and/or hydroxyl group at the C-3 position of the basic catechin structure, thereby improving the radical scavenging ability (62). Hence, catechins are potent antioxidants and play a protective role of dark tea in disease states related to reactive oxygen species (ROS). HSA showed significant positive correlation with TFs and TRs; no significant (*p* > 0.05) correlation was found between the four other *in vitro* antioxidant capacity assays and the two tea pigments. However, FRAP, ABTS, DPPH, and HSA had significant (*p* < 0.05) or highly significant (*p* < 0.01) negative correlation with TFs in dark tea in previous study (63). TFs was reported that can decrease the concentration of malondialdehyde and the activity of lactate acid dehydrogenase to improve superoxide dismutase capacity; as such, they are beneficial to increase the antioxidant activity (64). Moreover, colorless flavanols can be partially biotransformed into TFs and TRs under enzymatic oxidation by endogenous polyphenol oxidases and peroxidases, which contribute to the aroma and color of tea infusion (65). No significant (*p* > 0.05) correlation was observed between *in vitro* antioxidant activity and TBs (Figure 6). However, other reports noted that dark tea with higher TBs content exhibited stronger superoxide radical scavenging activity (54). Tea pigments are non-enzymatic scavengers that have effective free radical scavenging capacities and kinetics and are similar to polyphenols (66). Hence, TPC, TFC, GA, GC, EGC, catechin, EC, ECG, CG, and tea pigments contributed to the antioxidant capacities of FBT in this study.

Phenolic compounds, particularly gallic acid and catechins, exhibited greatly positive relationship with antioxidant activities in microbial fermented dark teas (e.g., Pu-erh tea, Fu brick tea, Liubao tea) (3, 14). Fungal communities influenced the metabolites of FBT during fermentation, thus leading to the variant antioxidant activity of FBT. Previous studies reported that TPC, TFC, and TFs are the main contributors to the strong antioxidant activity of dark tea (14). The TPC, TFC, and TFs in the ZJ sample is higher compared with SX, HN, and GZ samples might possibly be due to the higher abundance of *Monascus*, *Debaryomyces*, *Wallemia*, and *Quambalaria* (Figure 6), thus, ZJ sample was noted with stronger antioxidant activities than the SX, HN, and GZ samples. Furthermore, previous studies reported that GA has been considered to be one of the key antioxidants in dark tea (67), similar result was also found in this study. For instance, it was found that GA and all antioxidant assays were highly positively correlated (Figure 6), higher GA in ZJ and GX samples is one of an important reason for their higher antioxidant activity compared with the other three FBT. Besides, the characteristics of gallic acid, catechins, and tea pigments, which can act synergistically, additively or antagonistically should also be considered for the observed antioxidant activity of different FBT.

### Taste Characteristics of Different Fu Brick Tea Samples and Their Relationship to Metabolites

**FIGURE 9:**
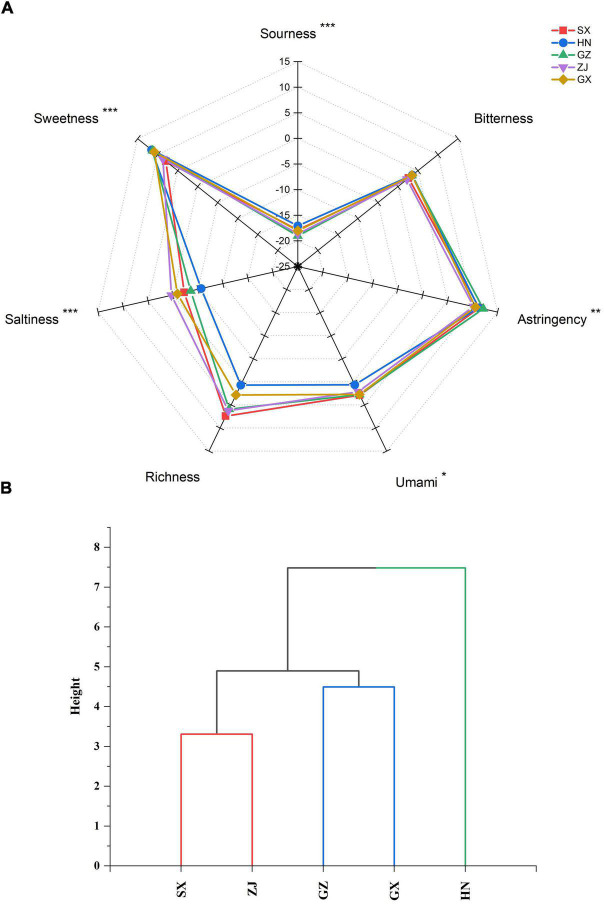
Spider plot **(A)** and dendrogram plot **(B)** of the taste characteristics of five Fu brick teas in different regions evaluated by E-tongue. *p < 0.05, **p < 0.01, ***p < 0.001

**FIGURE 10:**
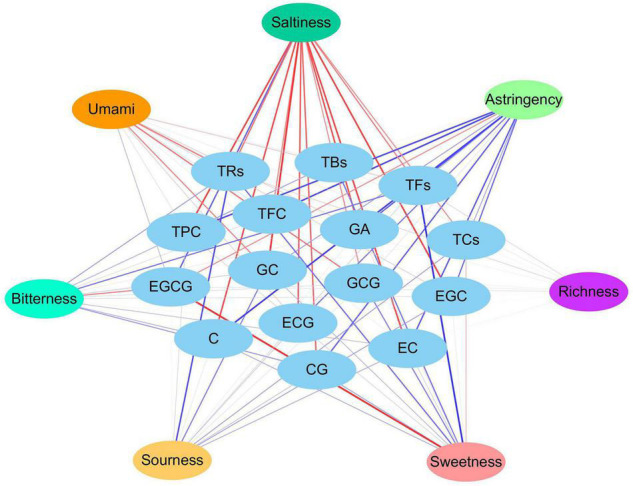
Network correlation analysis of the taste profile and the main metabolites.

Differences in the taste quality attributes of dark tea were investigated by E-tongue and presented in the spider plot (Figure 9A). The HN sample had the strongest sourness and sweetness; the ZJ sample had the strongest saltiness; the SX sample had the strongest umami; and the GZ sample had the strongest astringency among the FBT samples from different regions. No statistically significant difference (*p* > 0.05) in bitterness and richness was found among the FBT samples. The taste attributes of the FBT samples were clustered into three groups: HN, SX-ZJ, and GZ-GX (Figure 9B). The cluster of SX-ZJ samples demonstrated close taste characteristics. The GZ sample indicated similar taste attributes to the GX sample. The distinctive taste phenotype of FBT could be due to differences in the levels of various metabolites. Hence, network correlation analysis was performed to better understand the association between taste profiles and main constituents of dark tea (Figure 10). The bitterness of the FBT samples was positively related to EGCG (| *r*| = 0.576, *p* < 0.05) but negatively correlated with TFs (| *r*| = 0.616, *p* < 0.05), indicating that EGCG is the main contributor to the bitter taste of FBT. EGCG belongs to ester-type catechins and is thought to be the main source of bitterness in tea. The acceptance of green tea is related to bitterness genes, and that bitter taste is mainly linked with EGCG content (68), consistent with our research. Caffeine and several amino acids (arginine, alanine, etc.) also result in the bitter taste of tea infusion (8). Furthermore, the astringency of FBT had the most significant correlation with EGCG (| *r*| = 0.526 and *p* < 0.05). The amount of catechins in tea infusion greatly contributes to the bitterness and astringency of tea. However, previous studies reported that the level of catechins was sharply reduced under the action of enzymes during microbial fermentation (69). This finding may explain the decrease in bitterness and astringency and the increase in the mellow taste of FBT compared with green tea (8, 9, 25). The sweetness in tea was significantly (*p* < 0.05) negatively associated with GCG and tea pigments (TBs, TRs, and TFs). Sweet taste was related to soluble sugars including monosaccharides (most commonly glucose, galactose, fructose), polysaccharides, oligosaccharides, and a small number of other sugars (35). Sweetness is not the main taste of tea leaves, but it can harmonize the bitterness and astringency in tea soup. From our results, a negative relationship was found between sourness and the contents of GC and TRs. Tea contains a variety of organic acids (up to 3% of dry matter) mainly including GA, citric acid, ascorbic acid, malic acid, fatty acid, oxalic acid, etc. Most of the acid substances in tea are esterified with other compounds, such as alcohols, through processing, and the remaining part enters the tea soup to play a flavoring role (34). For example, soluble fruit acid is viscous and can increase the concentration of tea soup, making the tea taste richer and have better sense of hierarchy. Moreover, the main compounds responsible for the umami taste in tea infusion are amino acids such as theanine, glutamic acid, glutamine, aspartic acid, asparagine, etc., of which theanine and glutamate have the most remarkable effect (40). Interestingly, though the saltiness in FBT almost exhibited significant (*p* < 0.05) or highly significant (*p* < 0.01) positive correlations to all metabolites measured, the salty taste is mainly the result of the action of monovalent ions, such as potassium and sodium, and the corresponding negative ions, which are very low in content and are often masked by other tastes. Despite the lack of significant (*p* > 0.05) difference between richness and the 15 metabolites in our result, tea polyphenols and tea polysaccharides in tea infusion mainly contributed to richness (17). In fact, the taste characteristics of different FBT samples are not only depended by main metabolites catechins, tea polyphenols and tea pigments investigated in this study, some of other metabolites such as amino acids, soluble proteins, peptides, fatty acids, polysaccharides, etc. might also contributed to the taste attributes of FBT. These metabolites can act synergistically, additively or antagonistically with catechins to affect the taste attributes of FBT. Further research is essential to perform on identification of these compounds and their relationship with fungal community, as well as clarify their contribution to the taste characteristics of FBT.

## Conclusion

This study systematically characterized the diversity of fungal communities, chemical composition, antioxidant activity, and taste quality of FBT samples from five different regions of China. The relationship between bioactive metabolites and fungal communities and the key chemical substances contributing to the variations in antioxidant activity and taste profile were assessed. Results showed a total of 69 OTUs, which assigned into 5 phyla, 15 classes, 20 orders, 23 families, 27 genus, and 33 species, with *Eurotium* as predominant genus. The HN sample had the strongest fungal diversity and richness, followed by GX, and the ZJ sample had the lowest. Additionally, 15 metabolites (including total phenolics, total flavonoids, GA, 8 catechins, total catechins and 3 tea pigments) were evaluated. Higher contents of total phenolics, total flavonoids, TFs, TRs, catechin, EC, and CG were found in the ZJ sample. Higher levels of TBs and GCG were found in the SX sample. Higher contents of GA, GC, EGC, ECG, and total catechins were found in the GX sample. TPC, GA, EC, CG, and TFs were positively associated with most genera, while TFC, catechins, and TBs were negatively correlated. The antioxidant activities of FBT exhibited significant (*p* < 0.05) differences in the ABTS, DPPH, RP, FRAP, and HSA of the FBT samples from different regions. The GX sample had relatively stronger antioxidant capacity. The network correlation analysis uncovered that TPC, TFC, and most catechins contributed to the antioxidant activities in FBT. The taste phenotypes of FBT were classified into HN, SX-ZJ, and GZ-GX. The HN sample had the strongest sourness and sweetness; the ZJ sample showed the strongest saltiness; the SX sample showed the strongest umami; and the GZ sample showed the strongest astringency. This study provides insights into the fungal community in FBT and demonstrates the potential role of fungal community in metabolite formation to improve the quality characteristics of FBT products.

## Data Availability Statement

The raw data supporting the conclusions of this article will be made available by the authors, without undue reservation.

## Author Contributions

YC: methodology, writing—original draft, and investigation. JC: writing—original draft, data curation, and investigation. RC: methodology and investigation. XW, LH, and LX: investigation. ZL and YW: resources. ZL: conceptualization. MZ: supervision, methodology, and writing—review and editing. YX: project administration, supervision, funding acquisition, conceptualization, investigation, data curation, validation, formal analysis, methodology, writing—original draft, writing—review, and editing. All authors have read and agreed to the published version of the manuscript.

## Conflict of Interest

The authors declare that the research was conducted in the absence of any commercial or financial relationships that could be construed as a potential conflict of interest.

## Publisher’s Note

All claims expressed in this article are solely those of the authors and do not necessarily represent those of their affiliated organizations, or those of the publisher, the editors and the reviewers. Any product that may be evaluated in this article, or claim that may be made by its manufacturer, is not guaranteed or endorsed by the publisher.
