# Supplementary material for: Comparison of the Fungal Community, Chemical Composition, Antioxidant Activity, and Taste Characteristics of Fu Brick Tea in Different Regions of China
Source: Front Nutr. 2022 May 17;9:900138. doi: 10.3389/fnut.2022.900138 (PMC9152283; doi:10.3389/fnut.2022.900138)
Supplement: Supplementary file 1 [file Data_Sheet_1.docx]

Supplementary Material

# Supplementary Tables

**Supplementary Table 1.** Fungal community richness and diversity indices of five Fu brick teas in different regions.

| Sample ID | Reads | observed OTUs | ACE | Chao1 | Shannon | Simpson | Goods coverage |
| --- | --- | --- | --- | --- | --- | --- | --- |
| SX | 82,401±2,295^ab^ | 7.75±1.30^bc^ | 7.75±1.30^bc^ | 7.75±1.30^bc^ | 0.09±0.04^b^ | 0.02±0.01^b^ | 1.00 |
| HN | 85,183±794^a^ | 14.75±3.77^a^ | 14.75±3.77^a^ | 14.75±3.77^a^ | 0.91±0.47^a^ | 0.30±0.17^a^ | 1.00 |
| GZ | 84,494±1,453^ab^ | 9.50±2.18^abc^ | 9.81±2.69^abc^ | 9.50±2.18^abc^ | 0.12±0.13^b^ | 0.03±0.04^b^ | 1.00 |
| ZJ | 83,210±2,008^ab^ | 4.50±1.87^c^ | 6.28±1.62^c^ | 6.00±1.87^c^ | 0.03±0.02^b^ | 0.01±0.00^b^ | 1.00 |
| GX | 81,881±1,634^ab^ | 12.00±4.47^ab^ | 12.00±4.47^ab^ | 12.00±4.47^ab^ | 0.60±0.34^a^ | 0.24±0.15^a^ | 1.00 |

Letters indicate Duncan’s pairwise differences among different samples (*p* < 0.05).

## Supplementary Figures


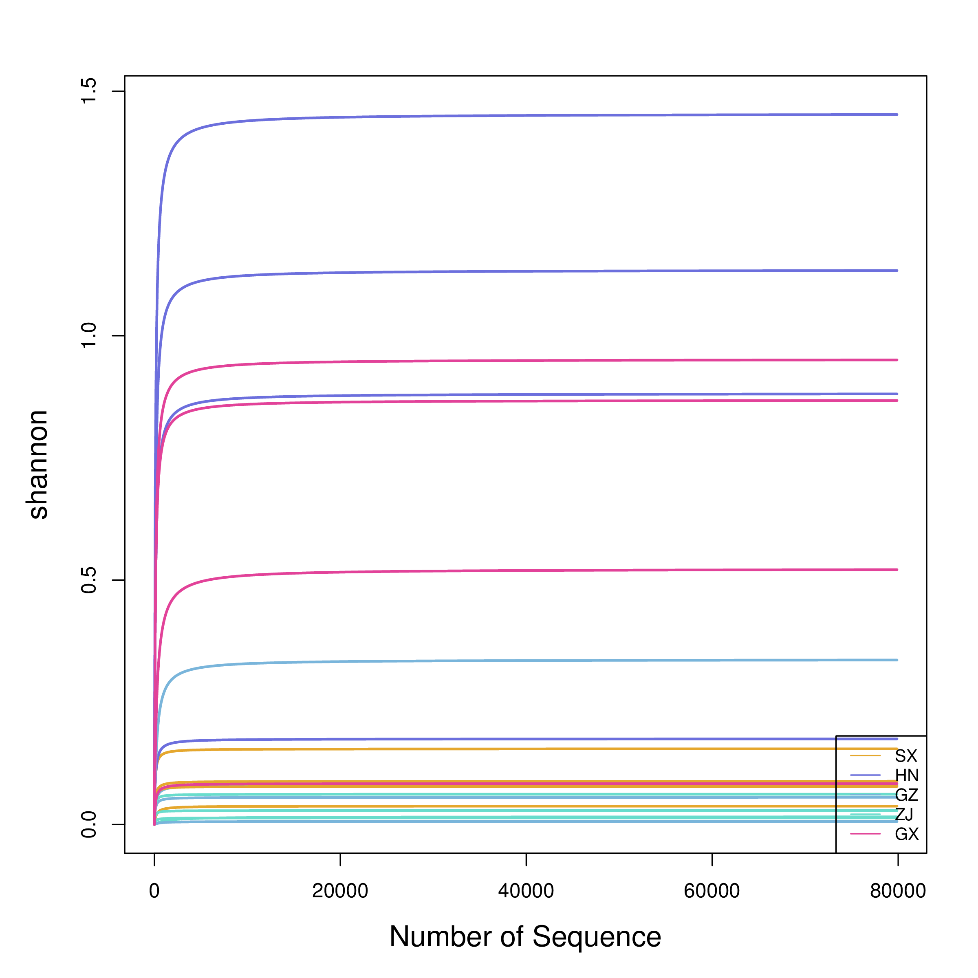


**Supplementary Figure 1.** The rarefaction curve for Shannon diversity of five Fu brick tea samples in different regions.


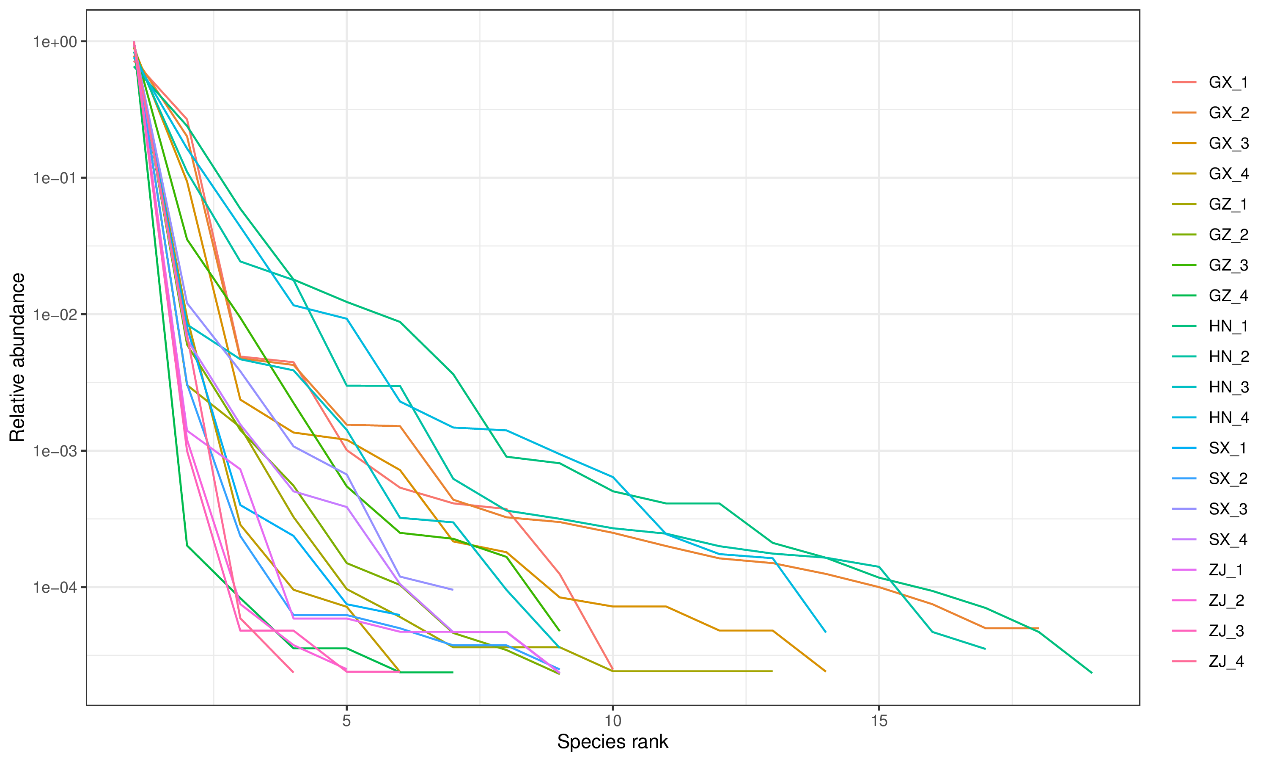


**Supplementary Figure 2.** Rank abundance curve of fungal OTUs derived from five Fu brick teas in different regions.


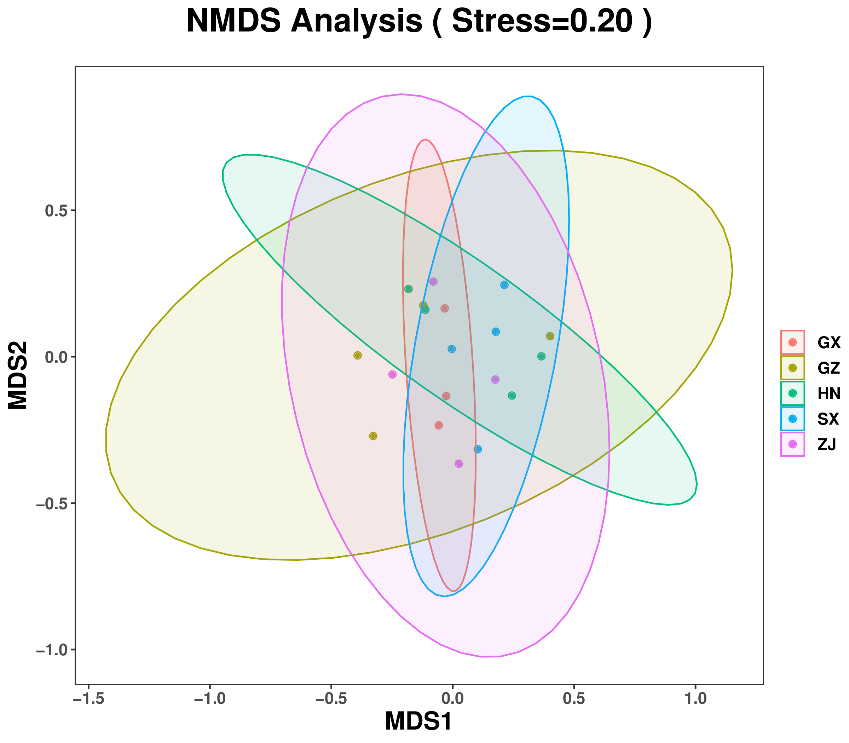


**Supplementary Figure 3.** The beta diversity NMDS results of fungal community compositions of five Fu brick tea samples in different regions.
